# Adropin as a Marker in Type 2 Diabetes: Insights Into Diabetic Kidney Disease and Chronic Heart Failure

**DOI:** 10.7759/cureus.91572

**Published:** 2025-09-03

**Authors:** Satyendra K Sonkar, Madhusudan Agrawal, Gyanendra K Sonkar, Vivek Bhosale, Medhavi Gautam, Akshay Pradhan, Satish Kumar, Deepak Bhagchandani, Abhishek Singh

**Affiliations:** 1 Medicine, King George's Medical University, Lucknow, IND; 2 Biochemistry, King George's Medical University, Lucknow, IND; 3 Toxicology and Experimental Medicine, Central Drug Research Institute, Lucknow, IND; 4 Nephrology, King George's Medical University, Lucknow, IND; 5 Cardiology, King George's Medical University, Lucknow, IND; 6 Community and Family Medicine, King George's Medical University, Lucknow, IND

**Keywords:** albuminuria, crp, egfr, hba1c, pro-bnp

## Abstract

Background: Type 2 diabetes mellitus (T2DM) is frequently associated with diabetic kidney disease (DKD) and chronic heart failure (CHF), conditions that share overlapping pathophysiological mechanisms. Adropin, a liver- and brain-derived peptide hormone, has emerged as a novel biomarker involved in metabolic regulation, endothelial function, and inflammation. However, its role in differentiating stages of T2DM-related complications remains underexplored.

Objectives: This study aimed to evaluate serum adropin levels in T2DM patients with and without DKD and to assess its association with coexisting CHF.

Methods: This was an observational case-control study which was conducted over a period of one year and included 111 participants divided into three groups: healthy controls, T2DM without nephropathy, and T2DM with nephropathy (with and without CHF). Serum adropin levels were measured using enzyme-linked immunosorbent assay (ELISA), and comprehensive clinical, biochemical, and echocardiographic assessments were done.

Results: Serum adropin levels were significantly reduced in T2DM patients (0.61 ± 0.13 ng/mL, p < 0.0001), with the lowest levels observed in those with both DKD and CHF (0.47 ± 0.12 ng/mL, p < 0.0001) compared to controls (0.76 ± 0.10 ng/mL, p < 0.0001). A progressive decline in adropin was noted with worsening glycemic control, renal dysfunction, chronic inflammation, and cardiac dysfunction. Significant inverse correlations were found between adropin and glycosylated hemoglobin (HbA1c) (*r* = -0.31), urinary albumin creatinine ratio (ACR) (*r* = -0.59), C - reactive protein (CRP) (*r* = -0.37), and N-terminal pro-B-type natriuretic peptide (NT-pro-BNP) (*r* = -0.44), while positive correlations existed with estimated glomerular filtration rate (eGFR) (*r* = +0.31). Receiver operating characteristic (ROC) curve analysis revealed high diagnostic accuracy for DKD (0.50 ng/mL, AUC  (area under curve) = 0.946), DKD with CHF (0.44 ng/mL, AUC = 0.965), and T2DM with CHF (0.54 ng/mL, AUC = 0.887).

Conclusion: The study demonstrated that serum adropin levels decline progressively with worsening cardiorenal-metabolic parameters. These findings reinforce the role of adropin as a sensitive marker of endothelial and metabolic dysfunction, especially in patients with coexisting T2DM, DKD, and CHF. Given its strong correlation with markers of glycemic control, renal impairment, and inflammation, adropin may serve as a noninvasive biomarker for early detection and risk stratification.

## Introduction

Type 2 diabetes mellitus (T2DM) is one of the most prevalent metabolic disorders worldwide, significantly contributing to morbidity and mortality through its complications, particularly chronic kidney disease (CKD) and chronic heart failure (CHF) [1,2]. These comorbidities often coexisted and shared overlapping pathophysiological mechanisms, including systemic inflammation, oxidative stress, neurohormonal dysregulation, and metabolic disturbances in multiple tissues [3,4]. Among microvascular complications, diabetic nephropathy (DN) remained the most common, affecting up to 40% of individuals with T2DM and serving as a leading cause of end-stage renal disease (ESRD) globally [5]. Its diagnosis and staging primarily relied on the presence of albuminuria and decline in glomerular filtration rate, with microalbuminuria representing the earliest clinical marker [6].

CHF, defined as the inability of the heart to maintain sufficient perfusion despite normal or elevated filling pressures, affected more than 64 million people globally. Its prevalence had been rising, largely due to population aging and the growing burden of T2DM [7]. The coexistence of CKD in CHF patients with T2DM complicated treatment decisions and reduced the diagnostic utility of conventional biomarkers such as natriuretic peptides [8]. These limitations highlighted the need to explore novel biomarkers capable of reflecting the interplay between renal and cardiac dysfunction in diabetes.

Adropin, a peptide hormone secreted primarily by the liver and brain, emerged as a potential biomarker with roles in energy regulation, glucose metabolism, and endothelial function. It exerted its actions via activation of vascular endothelial growth factor receptor 2 (VEGFR2) and glucose transporter type 4 (GLUT4), contributing to improved vascular integrity and glucose utilization [9]. Additionally, adropin downregulated inflammatory cytokines and improved lipid metabolism, myocardial efficiency, and coronary perfusion [10].

Studies had reported that serum adropin levels were reduced in obese and diabetic individuals and were inversely associated with renal dysfunction in T2DM [11]. Conversely, elevated adropin levels were linked to reduced atherosclerotic burden and improved vascular outcomes in diabetic patients [12]. Interventions such as sodium-glucose cotransporter 2 (SGLT2) inhibitors, sitagliptin therapy, and aerobic exercise had been shown to raise serum adropin levels and improve cardiometabolic profiles [13-16]. Although elevated adropin levels were observed in acute HF and varied with New York Heart Association (NYHA) class, their ability to differentiate between DN and CHF in the setting of T2DM remained uncertain [17].

The present study aimed to assess serum adropin levels in T2DM patients with and without DN and examine its association with CHF. A better understanding of this relationship could support the development of adropin as a biomarker for early detection and risk stratification in patients with coexisting T2DM, diabetic kidney disease (DKD), and CHF.

## Materials and methods

This case-control observational study was conducted at a tertiary care academic center in Northern India. The study duration spanned one year. Ethical clearance was obtained from the Institutional Ethics Committee, and informed written consent was taken from all participants prior to enrolment.

The sample size was determined based on previously published prevalence data and study feasibility. After adjusting for potential data loss, a total of 111 participants were included in the study. Participants were allocated into three groups, each comprising 37 individuals. Group A consisted of controls without T2DM, DN, or CHF. Group B included patients of T2DM, with or without CHF and with no evidence of nephropathy. Group C included patients of DKD, with or without CHF. Inclusion criteria were adults aged between 18 and 65 years who were willing and able to provide informed consent. Participants were excluded if they had acute on chronic liver or renal disease, acute or acute-on-chronic heart failure, malignancy, known inflammatory conditions, or active infections.

All participants underwent a comprehensive clinical evaluation, which included history taking, physical examination, and recording of demographic details, body mass index (BMI), blood pressure, duration of diabetes, medication history, and lifestyle factors such as smoking and alcohol use.

DKD patients had an estimated glomerular filtration rate (eGFR) of less than 60 ml/min/1.73 m² calculated using the CKD-EPI creatinine equation, and an elevated urine albumin-to-creatinine ratio (ACR) measured from a spot urine sample. ACR values of 30-299 mg/g indicated microalbuminuria, while values ≥300 mg/g indicated macroalbuminuria.

The diagnosis of CHF was based on history of clinical signs and symptoms and confirmed through two-dimensional echocardiography. Classification was done according to the European Society of Cardiology guidelines 2023 (Table 1).

**Table 1:** Diagnostic criteria of chronic heart failure according to the European Society of Cardiology guidelines 2023 HF: heart failure; HFmrEF: heart failure with mildly reduced ejection fraction; HFpEF: heart failure with preserved ejection fraction; HFrEF: heart failure with reduced ejection fraction; LV: left ventricle; LVEF: left ventricular ejection fraction Symptoms include breathlessness, orthopnea, paroxysmal nocturnal dyspnea, reduced exercise tolerance, fatigue, tiredness, increased time to recover after exercise, and ankle swelling. Signs includes elevated jugular venous pressure, hepatojugular reflux, third heart sound (gallop rhythm), and laterally displaced apical impulse

Type of HF		HFrEF	HFmrEF	HFpEF
Criteria	1	Symptoms ± signs	Symptoms ± signs	Symptoms ± signs
	2	LVEF ≤40%	LVEF 41-49%	LVEF ≥50%
	3	–	–	Objective evidence of cardiac structural and/or functional abnormalities consistent with the presence of LV diastolic dysfunction/raised LV filling pressures, including raised natriuretic peptides

All participants underwent a standardized set of laboratory investigations. These included hematological tests such as complete blood count; renal function tests including serum urea, creatinine, sodium, potassium, and eGFR; liver function tests such as total bilirubin, serum glutamic-oxaloacetic transaminase (SGOT) and serum glutamic-pyruvic transaminase (SGPT), alkaline phosphatase, total protein, and albumin; glycemic profile including fasting and postprandial plasma glucose and HbA1c; and a lipid profile including total cholesterol, low-density lipoprotein (LDL), high-density lipoprotein (HDL), and triglycerides. C-reactive protein (CRP) as a maker of inflammation and N-terminal pro-B-type natriuretic peptide (NT-pro-BNP) for assessing cardiac function. Urine ACR was measured using the immunoturbidimetric method from a mid-stream spot sample.

Cardiac evaluation was performed using electrocardiography and two-dimensional echocardiography to assess parameters such as left ventricular ejection fraction (LVEF), chamber dimensions, and valvular status.

For serum adropin estimation, fasting venous blood samples (5 mL) were collected in serum separator tubes under aseptic conditions. Samples were allowed to clot at room temperature and then centrifuged at 1000 × g for 20 minutes. The separated serum was aliquoted into labeled microcentrifuge tubes and stored at -20°C until further analysis. Adropin levels were quantified using a commercially available enzyme-linked immunosorbent assay (ELISA) kit specific to human adropin with detection range from 31.25 pg/mL to 2000 pg/mL (sensitivity: 13.7 pg/mL), and all assays were run in duplicates to ensure reliability. Care was taken to avoid hemolysis and repeated freeze-thaw cycles during the handling of serum samples.

Statistical analysis was conducted using SPSS version 26.0IBM SPSS Statistics for Windows, Version 26 (Released 2018; IBM Corp., Armonk, New York, United States. Continuous variables were expressed as mean ± standard deviation and compared using one-way analysis of variance (ANOVA) with post hoc Tukey tests or independent t-tests, as appropriate. Categorical variables were analyzed using the Chi-square or Fisher’s exact test. Correlations between serum adropin and clinical parameters were assessed using Pearson or Spearman correlation coefficients. Diagnostic performance of serum adropin for detecting DN and CHF was evaluated using receiver operating curve receiver operating characteristic (ROC) curve analysis, with calculation of AUC, optimal cut-off values, sensitivity, specificity, predictive values, and overall accuracy. A p-value of <0.05 was considered statistically significant.

## Results

The mean age was significantly higher in the disease groups compared to controls (p < 0.0001), with a male preponderance; however, sex distribution was uniform across the groups (p > 0.3). BMI was comparable among all groups (p > 0.05) (Table 2).

**Table 2:** Comparison of various parameters across groups BMI: body mass index; T2DM: type 2 diabetes mellitus; DKD: diabetic kidney disease; HbA1c: glycosylated hemoglobin; eGFR: estimated glomerular filtration rate; LDL: low-density lipoprotein; HDL: high-density lipoprotein; CRP: C-reactive protein; NT-pro-BNP: N-terminal pro-B-type natriuretic peptide

Parameter	Reference range (unit)	Group A (control) (n = 37)	Group B (T2DM without nephropathy) (n = 37)	Group C (DKD) (n = 37)	p-value
Age	18-65 (years)	39.35 ± 15.38	54.35 ± 12.64	59.08 ± 10.21	<0.001
Male	-	70.3	67.6	62.2	>0.05
BMI	18.5-24.9 (kg/m2)	24.3 ± 4.34	24.83 ± 3.60	24.54 ± 2.69	>0.05
Systolic blood pressure	120-128 (mmHg)	121.1 ± 4.80	137.5 ± 23.46	134.1 ± 24.31	<0.001
Fasting glucose	70-100 (mg/dL)	92.45 ± 8.10	158.73 ± 26.90	174.62 ± 31.85	<0.001
HbA1c	4.0-5.6 (% )	5.16 ± 0.51	9.63 ± 2.19	7.74 ± 1.44	<0.001
eGFR	≥60 (ml/min/1.73m2)	94.04 ± 7.01	80.84 ± 20.37	24.24 ± 13.74	<0.001
Total Cholesterol	<200 (mg/dL)	155.09 ± 46.04	187.84 ± 67.61	149.14 ± 56.86	>0.05
LDL	<70 (mg/dL)	80.38 ± 34.01	104.72 ± 46.93	90.97 ± 47.35	>0.05
Triglycerides	<150 (mg/dL)	177.23 ± 78.38	213.16 ± 121.90	155.05 ± 58.46	>0.05
HDL	>50 (mg/dL)	54.9 ± 8.16	49.2 ± 3.35	45.0 ±3.77	>0.05
CRP	0-6(mg/L)	3.69 ± 1.27	33.34 ± 30.19	48.79 ± 38.72	<0.001
NT-pro-BNP	0-125 (pg/mL))	106.24 ± 32.54	685.12 ± 130.13	1069 ± 159.6	<0.001

Systolic blood pressure was significantly elevated in diabetic subjects, while it remained within normal limits in the control group (p < 0.001) (Table 2). Approximately 60% of patients in Group B (T2DM without nephropathy) and 80% in Group C (DKD) were hypertensive.

Glycemic parameters were markedly altered in the diabetic groups. Fasting blood sugar levels were 174.62 ± 31.85 mg/dL in Group C (DKD) and 158.73 ± 26.90 mg/dL in Group B (T2DM without nephropathy), with a statistically significant difference (p < 0.0001). HbA1c followed a similar trend, being significantly elevated at 9.63 ± 2.19% in Group B and 7.74 ± 1.44% in Group C (Table 2). Approximately 70% of patients in Group B and 50% in Group C had uncontrolled blood sugar levels (Table 2).

Lipid profile assessment in Group B revealed significant dyslipidemia, with elevated total serum cholesterol (187.84 ± 67.61 mg/dL), LDL (104.72 ± 46.93 mg/dL), and triglycerides (213.16 ± 121.90 mg/dL) (p < 0.05) (Table 2), whereas HDL and very low-density lipoprotein (VLDL) levels did not show statistically significant differences.

In the DKD group (Group C), renal function was severely impaired, with a mean eGFR of 24.24 ± 13.74 mL/min/1.73 m² (p < 0.0001). Among Group C participants, 51.4% had microalbuminuria and 48.6% had macroalbuminuria. The inflammatory marker CRP was markedly elevated in both Group B (33.34 ± 30.19 mg/dL) and Group C (48.79 ± 38.72 mg/L). Cardiac biomarker NT-pro-BNP levels were also significantly higher in both Group B and Group C (p < 0.001) (Table 2). In the T2DM with CHF cohort comprising 17 patients, 5.9% were having heart failure with preserved ejection fraction (HFpEF), 52.9% with mildly reduced ejection fraction (HFmrEF), and 41.2% with reduced ejection fraction (HFrEF). Among the 17 patients in the DKD with CHF cohort, 58.8% had HFmrEF and 41.2% had HFrEF, with no cases of HFpEF.

Serum adropin levels were significantly reduced in all disease groups compared to controls. The lowest levels were observed in patients with DKD and CHF (0.47 ± 0.12 ng/mL), followed by DKD without CHF (0.54 ± 0.12 ng/mL), T2DM with CHF (0.54 ± 0.13 ng/mL), and T2DM without CHF (0.67 ± 0.09 ng/mL) (Table 3). The decline in serum adropin levels was statistically significant across all comparisons with the control group (p < 0.0001). ROC analysis revealed that serum adropin exhibited excellent diagnostic accuracy in differentiating disease groups from controls. The AUC was 0.946 for distinguishing DKD from controls, 0.965 for DKD with CHF, and 0.887 for T2DM with CHF. The optimal cut-off values for serum adropin were 0.54 ng/mL, 0.44 ng/mL, and 0.59 ng/mL, respectively. These thresholds provided sensitivities of over 83%, 97%, and 94%, along with specificities of 81%, 70%, and 70%, respectively.

**Table 3:** Groupwise adropin levels: distribution T2DM: type 2 diabetes mellitus; CHF: chronic heart failure; DKD: diabetic kidney disease

Parameter	Group A (control) (n = 37) (mean ± SD)	Group B T2DM (n = 37) (mean ± SD)			Group C DKD (n = 37) (mean ± SD)			p-value
S. adropin (ng/ml)	0.76 ± 0.10	0.61 ± 0.13			0.50 ± 0.12			<0.0001
		T2DM without CHF (n = 20)	T2DM with CHF (n = 17)	p-value	DKD without CHF (n = 20)	DKD with CHF (n = 17)	p-value	
		0.67 ± 0.09	0.54 ± 0.13	<0.0001	0.54 ± 0.12	0.47 ± 0.12	<0.0001	

Pearson correlation analysis showed a significant inverse relationship between serum adropin and markers of inflammation, glycemic burden, and renal impairment. Strongest negative correlations were found with urine ACR (r = -0.59, p < 0.0001), pro-BNP (r = -0.44, p < 0.0001), CRP (r = -0.37, p < 0.0001), and HbA1c (r = -0.31, p = 0.0008). Conversely eGFR (r = +0.31, p = 0.0008) showed significant positive associations with adropin. These correlations are visually summarized in Table 4, underscoring the peptide’s link with metabolic, renal, and cardiovascular parameters.

**Table 4:** Pearson index correlation with various clinical parameters BMI: body mass index; ACR: albumin creatinine ratio; HbA1c: glycosylated hemoglobin; eGFR: estimated glomerular filtration rate; LDL: low-density lipoprotein; CRP: C-reactive protein; pro-BNP: pro B-type natriuretic peptide

S. adropin level	Pearson r	95% confidence interval	p-value
Hemoglobin	0.31	0.13 to 0.47	0.0011*
BMI	-0.09	-0.27 to 0.09	0.33
Urine ACR	-0.59	-0.70 to -0.45	<0.0001*
HbA1C	-0.31	-0.47 to -0.13	0.0008*
CRP	-0.37	-0.52 to -0.19	<0.0001*
PRO-BNP	-0.44	-0.57 to -0.28	<0.0001*
eGFR	0.31	0.13 to 0.47	0.0008*
LDL	-0.22	-0.40 to -0.03	0.021*

Collectively, these findings confirm that serum adropin levels decline progressively with worsening glycemic control, renal dysfunction, and heart failure and demonstrate potential utility as a biomarker for identifying early nephropathy and cardiovascular compromise in T2DM patients.

## Discussion

This study demonstrated a clear and statistically significant reduction in serum adropin levels among patients with T2DM, with further declines observed in those with concurrent DKD and CHF, compared to healthy controls. This trend suggests a progressive suppression of this hepatokine in association with worsening metabolic and cardiorenal complications. These findings align with previous reports indicating decreased serum adropin levels in diabetic patients, especially those with complications such as nephropathy or CHF, reinforcing its role as a peptide sensitive to metabolic stress [16,18-20].

In our study, the average BMI was comparable across all groups. However, previous studies have demonstrated an inverse correlation between serum adropin and BMI. For instance, Berezina et al. reported a correlation coefficient of r = -0.29 and Indian study by Shah et al. showed similar relation r = -0.29 [16,18]. This inverse relationship is further supported by a study on patients undergoing bariatric surgery, where serum adropin levels were significantly higher six months post-surgery compared to pre-surgery levels [21]. These findings suggest that adiposity significantly influences adropin levels and may play an important role in the pathophysiology of diabetes.

Adropin levels were significantly lower in diabetic patients without heart failure and nephropathy than controls (0.67 ± 0.09 vs 0.76 ± 0.10 ng/mL). Similar trends were reported by Berezina et al. (4.15 vs 5.88 ng/mL), Shah et al. (2.12 ± 0.12 vs 3.82 ± 0.40 ng/mL), and Es-Haghi et al. (3.35 ± 0.36 vs 4.21 ± 0.52 ng/mL), consistent with a meta-analysis done previously in wide diverse population [16,18-20]. Since its discovery, adropin has been linked to glucose homeostasis, prompting numerous investigations into the mechanisms underlying its regulation. For example, in streptozotocin-induced diabetic rats, hyperglycemia was associated with elevated adropin expression and activation of the signal transducer and activator of transcription 3 (STAT3) in the liver, suggesting a regulatory mechanism for increased adropin levels and ENHO gene expression [22]. In skeletal muscle, adropin has been shown to play a critical role in modulating glucose metabolism, particularly in diet-induced obese (DIO) mice with insulin resistance [23]. It enhances glucose oxidation while suppressing fatty acid oxidation, resulting in increased glucose uptake and improved mitochondrial function. These metabolic effects are mediated through the suppression of peroxisome proliferator-activated receptor gamma coactivator-1α (PGC-1α), a key regulator of genes involved in fatty acid oxidation. Additionally, adropin enhances skeletal muscle sensitivity to insulin by promoting insulin-induced Akt phosphorylation and increasing the cell surface expression of glucose transporter 4 (GLUT4) [23].

Our study found a significant inverse correlation between serum adropin and levels and the glycemic burden marker HbA1c (r = -0.31), aligning with prior reports: Es-Haghi et al. (r = -0.24), Shah et al. (r = -0.30), and Berezina et al. (r = -0.73) [16,18,20]. These findings, including our own, collectively reinforce the inverse relationship between adropin levels and long-term glycemic control in T2DM. Earlier research has also demonstrated that treatments such as SGLT2 inhibitors, sitagliptin, and lifestyle interventions like increased physical activity, which are known to improve glycemic control, were associated with elevated adropin levels in newly diagnosed T2DM patients [13-15]. These findings suggest that adropin may not only serve as a biomarker but could also be modifiable through therapeutic interventions also established in experimental studies [24].

Persistent hyperglycemia is a well-recognized contributor to the development of microvascular complications in T2DM, including DKD. Serum adropin levels were significantly lower in DKD patients, consistent with prior studies. Hu et al. reported 2.73 vs. 3.17 ng/mL (p < 0.001), and Es-Haghi et al. found 2.77 ± 0.36 vs. 3.35 ± 0.36 ng/mL in DKD vs. non-DKD T2DM patients [11,16,20]. Serum adropin levels showed a significant correlation with markers of renal dysfunction across all groups, specifically, a negative correlation with the urinary ACR (r = -0.59) and a positive correlation with eGFR (r = 0.31). These findings align with prior studies: Hu et al. reported an inverse correlation between adropin and ACR (r = -0.35) and a positive one with eGFR (r = 0.173) [11]; Berezina et al. found a similar eGFR association (r = 0.30) [16]; and Es-Haghi et al. reported a strong negative correlation with ACR (r = -0.711) [20]. Collectively, these results suggest a consistent association between lower adropin levels and declining renal function in patients with T2DM. These findings support the potential involvement of adropin in renal function. The underlying pathogenesis may be linked to adropin’s role in maintaining endothelial integrity and exerting anti-inflammatory effects. Prior research has demonstrated adropin expression in rat kidney tissue, including the glomerulus, peritubular interstitial cells, and peritubular capillary endothelial cells [25]. Although the exact mechanism by which adropin influences DKD remains unclear, studies have shown that adropin significantly reduces mRNA expression levels of pro-inflammatory cytokines such as tumor necrosis factor-alpha (TNF-α) and interleukin-6 (IL-6) in the pancreatic tissue of diabetic rats [24]. Based on these observations, we hypothesize that adropin may have a protective role in the progression of DN through its anti-inflammatory actions.

There is substantial evidence linking increased cardiovascular disease burden with both diabetes and renal dysfunction. As noted, serum adropin levels were significantly lower in CHF patients. T2DM with CHF had lower levels than those without (0.54 ± 0.13 vs. 0.67 ± 0.09 ng/mL, p < 0.0001), and DKD with CHF showed further decline compared to DKD without CHF (0.47 ± 0.12 vs. 0.54 ± 0.12 ng/mL, p < 0.0001). Consistent with Berezina et al., adropin levels were lower in T2DM patients without HF/CKD (4.15 ng/mL) than in healthy controls (5.88 ng/mL, p = 0.012), and even lower in T2DM with HF (2.37 ng/mL, p = 0.001). Among T2DM-HF patients, those with CKD had the lowest levels (2.08 vs. 2.65 ng/mL, p = 0.001)[16].

Furthermore, NT-proBNP, a well-established biomarker of heart failure, showed a significant negative correlation with serum adropin levels (r = -0.44) in our study. This finding contrasts with previous reports, where a positive correlation between adropin and NT-proBNP was observed, such as r = 0.36 by Berezina et al. [16] and r = 0.72 by Lian et al. [26]. Adropin immunoreactivity has been detected in various tissues, including all three layers of the heart [27]. In diabetic patients, elevated cardiac fatty acid oxidation and impaired insulin signaling are known to reduce cardiac efficiency and contribute to the development of cardiovascular complications [28]. Adropin is thought to exert cardioprotective effects [19], in part by enhancing endothelial function through increased nitric oxide (NO) production. This occurs via activation of endothelial NO synthase (eNOS), and studies have shown that the use of specific inhibitors for Akt and Erk1/2 blocks eNOS activation by adropin, indicating that adropin-induced NO production is dependent on Akt and Erk1/2 pathways. Furthermore, adropin appears to regulate eNOS bioactivity through upstream stimulation of VEGFR2, a key receptor involved in endothelial cell function and angiogenesis. This activation of the PI3K-Akt and ERK1/2 signaling pathways ultimately contributes to improved cardiac perfusion and vascular health [9].

The pathophysiological basis for adropin suppression in conditions such as diabetes, DKD, and CHF may be attributed to its regulatory roles in glucose metabolism, endothelial NO availability, and inflammation modulation. Previous studies have demonstrated that adropin enhances insulin signaling through GLUT4 translocation and activation of the PI3K-Akt pathway, while also exerting anti-inflammatory effects by downregulating proinflammatory cytokines such as TNF-α, IL-6, and CRP [9,10]. In alignment with these observations, our study also identified a significant inverse relationship between serum adropin levels and CRP, a key marker of systemic inflammation (r = -0.37). His finding reinforces the potential anti-inflammatory role of adropin and is comparable to results reported by Berezina et al., who documented a similar negative correlation (r = -0.28) [16].

Additionally, our findings underscore the potential diagnostic utility of serum adropin. ROC analysis revealed excellent discriminatory power for identifying DKD (AUC = 0.946), DKD with CHF (AUC = 0.965), and T2DM with CHF (AUC = 0.887). The optimal serum adropin cut-off values were 0.54 ng/mL, 0.44 ng/mL, and 0.59 ng/mL, respectively, corresponding to sensitivities of 83%, 97%, and 94%, and specificities of 81%, 70%, and 70%. Comparable results were reported by Berezina et al., who identified an optimal adropin threshold of 2.3 ng/mL for DKD, with an AUC of 0.86 and sensitivity and specificity exceeding 75% [16]. Similarly, Es-Haghi et al. determined a cut-off value of 3.2 ng/mL for distinguishing T2DM patients without renal dysfunction, with an AUC of 0.83, sensitivity of 80%, and specificity above 60% [20]. However, it is important to emphasize that a standardized reference range for serum adropin in humans has yet to be established, which may contribute to variability across studies.

Taken together, the present study reaffirms and expands upon existing evidence by showing that adropin is significantly lower in patients with T2DM, especially those with cardiorenal complications. Its strong correlations with eGFR, ACR, CRP, and PRO-BNP indicate its potential utility not only in diagnosis but also in risk stratification and longitudinal monitoring. This also raises the possibility of using adropin as a therapeutic target, considering evidence that pharmacologic agents and lifestyle interventions can modulate its levels [13-16].

Limitations

A large, multicentric, prospective study is essential to validate the findings of the current research. Establishing a standardized reference value for adropin is necessary before it can be adopted as a universal biomarker for T2DM and its complications. Subgrouping diabetic patients with and without nephropathy further into those with and without CHF may have diluted disease-specific effects. Additionally, potential confounding factors such as diet, physical activity, medication use, and other variables were not controlled for in this study.

## Conclusions

This study provided compelling evidence that serum adropin levels were significantly reduced in patients with T2DM, with the lowest concentrations observed in those suffering from both DKD and CHF. This reflects the increasing burden of metabolic, renal, and cardiac dysfunction. Adropin was inversely related to hyperglycemia (uncontrolled blood glucose), with chronic inflammation arising due to various oxidative stress and endothelial dysfunction. In patients with DKD, the preclinical stage of microalbuminuria correlated with adropin levels, and with worsening albuminuria, adropin levels further decreased. The low level of adropin in diabetic patients with CHF suggests that it may have a cardioprotective effect due to its vasodilatory properties, leading to improved cardiac perfusion and vascular health. Anemia aggravates these cardiometabolic complications.

The above findings highlight adropin’s potential not only as a diagnostic and prognostic biomarker and may be also as a modifiable factor responsive to lifestyle as well as therapeutic interventions. With further validation in large-scale, prospective studies, adropin could be incorporated into clinical practice for early detection, improved risk stratification, and more personalized management of T2DM and its cardiorenal complications.
